# Supplementary material for: Multi-cancer analysis of histopathologic MSI screening based on digital histology image
Source: PLoS One. 2025 Sep 15;20(9):e0332034. doi: 10.1371/journal.pone.0332034 (PMC12435642; doi:10.1371/journal.pone.0332034)
Supplement: S1 File — (ZIP) [file pone.0332034.s001.zip › Supporting_Information/S1_Fig.pdf]

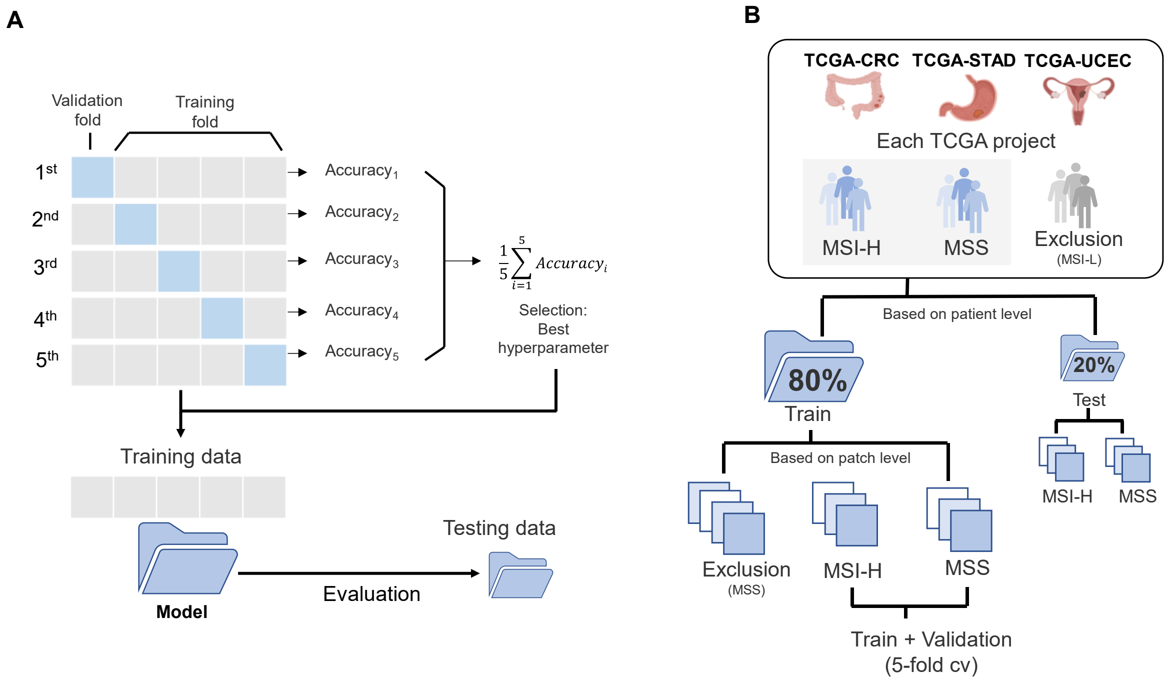

S1 Fig. Evaluation procedure and dataset division for tumor and MSI classifier models. **A**. The evaluation procedure using k-fold cross validation of the tumor and MSI classifier model. **B**. Dataset division for the MSI classification model
