# Supplementary material for: Multi-cancer analysis of histopathologic MSI screening based on digital histology image
Source: PLoS One. 2025 Sep 15;20(9):e0332034. doi: 10.1371/journal.pone.0332034 (PMC12435642; doi:10.1371/journal.pone.0332034)
Supplement: S1 File — (ZIP) [file pone.0332034.s001.zip › Supporting_Information/S1_Table.pdf]

S1 Table. Detailed model structure.

Model Name	Architecture	Type	Fully connected layers	Trainable parameters
EfficientNetb0 Model1	EfficientNetb0	Model1	-	2,084,370
EfficientNetb0 Model2	EfficientNetb0	Model2	2 hidden layers	789,250
EfficientNetb0 Model3	EfficientNetb0	Model3	2 hidden layers	2,871,058
ResNet18 Model1	ResNet18	Model1	-	8,394,754
ResNet18 Model2	ResNet18	Model2	1 hidden layer	132,354
ResNet18 Model3	ResNet18	Model3	1 hidden layer	8,526,082
VGG19 Model1	VGG19	Model1	-	124,273,666
VGG19 Model2	VGG19	Model2	4 hidden layers	11,852,314
VGG19 Model3	VGG19	Model3	4 hidden layers	136,117,786
ConvNeXt Model1	ConvNeXt	Model1	-	9,529,346
ConvNeXt Model2	ConvNeXt	Model2	3 hidden layers	527,106
ConvNeXt Model3	ConvNeXt	Model3	3 hidden layers	10,053,378
NAT Model1	NAT	Model1	-	5,262,626
NAT Model2	NAT	Model2	3 hidden layers	395,522
NAT Model3	NAT	Model3	3 hidden layers	5,656,098

Model1: The pre-trained base model with some trainable layers. **Model2**: The pre-trained base model with fronzen layers includes several additional hidden layer, incorporating dropout and batch normalization. **Model3**: The pre-trained base model with some trainable layers includes several additional hidden layer, incorporating dropout and batch normalization
