# Supplementary material for: Multi-cancer analysis of histopathologic MSI screening based on digital histology image
Source: PLoS One. 2025 Sep 15;20(9):e0332034. doi: 10.1371/journal.pone.0332034 (PMC12435642; doi:10.1371/journal.pone.0332034)
Supplement: S1 File — (ZIP) [file pone.0332034.s001.zip › Supporting_Information/S2_Fig.pdf]

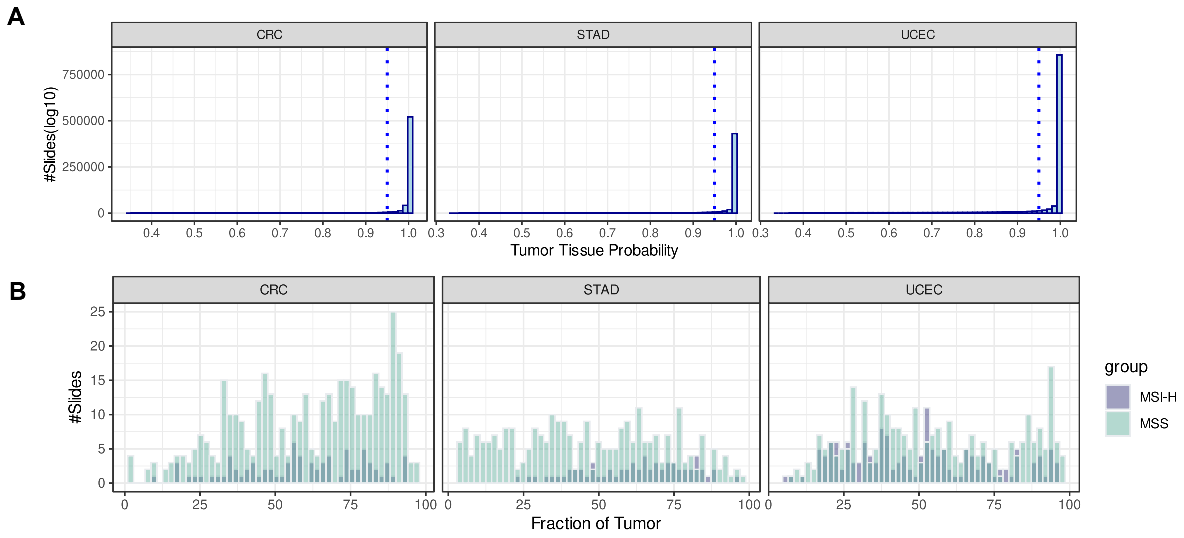

S2 Fig. Tumor tissue probability and distribution per slide. A. A histogram depicting the probability of tumor tissue at the patch level is shown, with the blue line indicating the cut-off value (0.95) for selecting tumor tissue. **B**. Distribution of tumor fraction per slide.
