# Supplementary figures and images for: Multi-cancer analysis of histopathologic MSI screening based on digital histology image
Source: PLoS One. 2025 Sep 15;20(9):e0332034. doi: 10.1371/journal.pone.0332034 (PMC12435642; doi:10.1371/journal.pone.0332034)

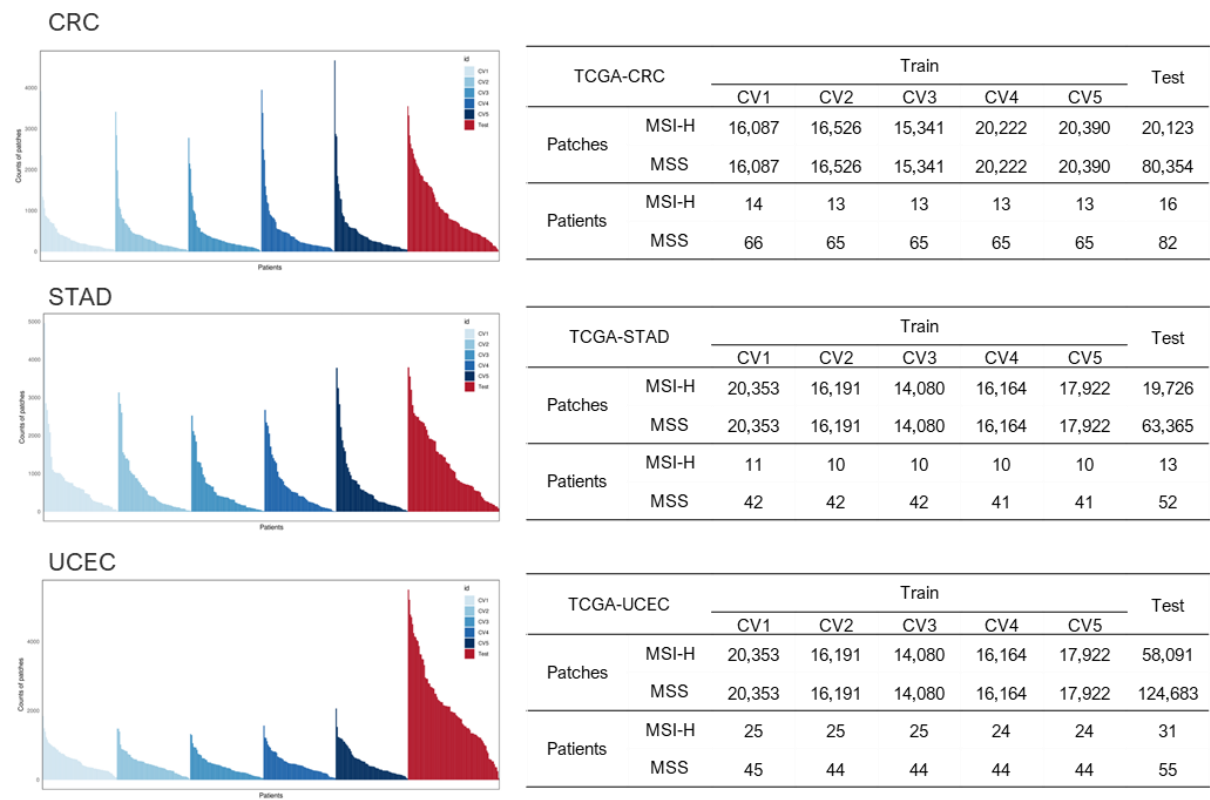

**S3 Fig. Datasets for train and test for the MSI classifier.**

Supplement: S1 File — (ZIP) [file pone.0332034.s001.zip › Supporting_Information/S3_Fig.pdf]

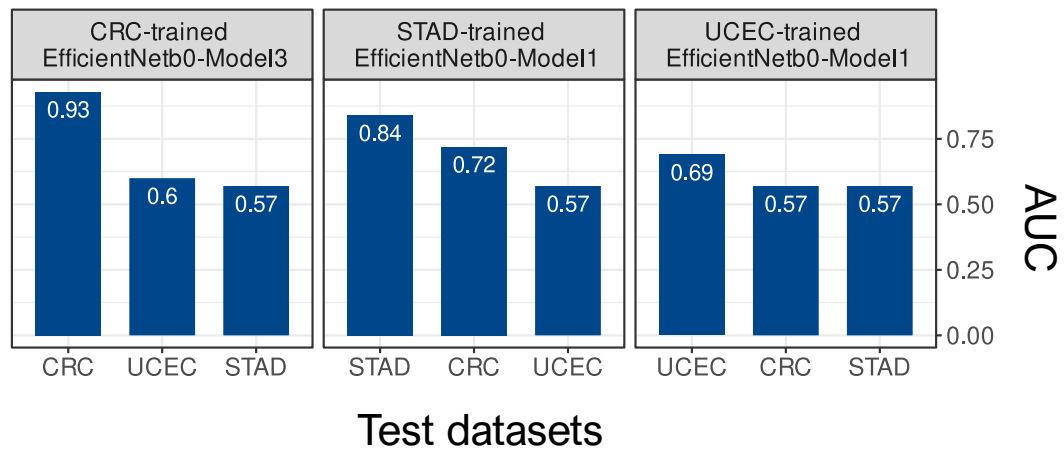

**S4 Fig. Comparing performances between the corresponding and cross tissue trained models.**

Supplement: S1 File — (ZIP) [file pone.0332034.s001.zip › Supporting_Information/S4_Fig.pdf]

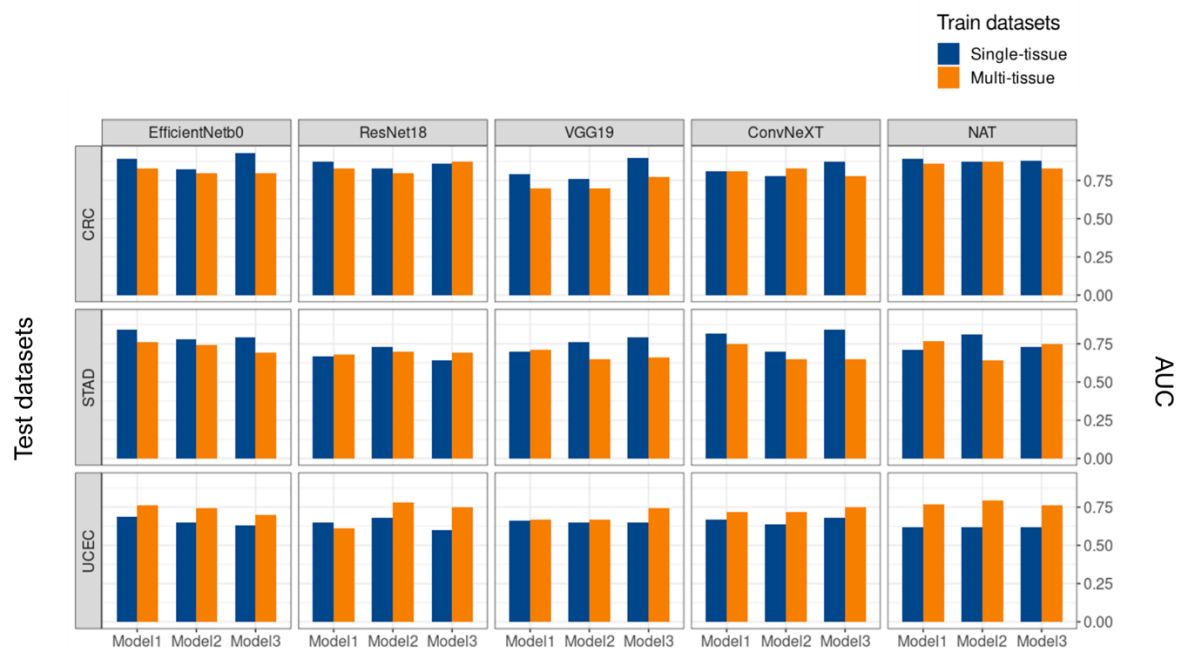

**S5 Fig. Comparing performances between single-tissue and multi-tissue trained models.**

Supplement: S1 File — (ZIP) [file pone.0332034.s001.zip › Supporting_Information/S5_Fig.pdf]

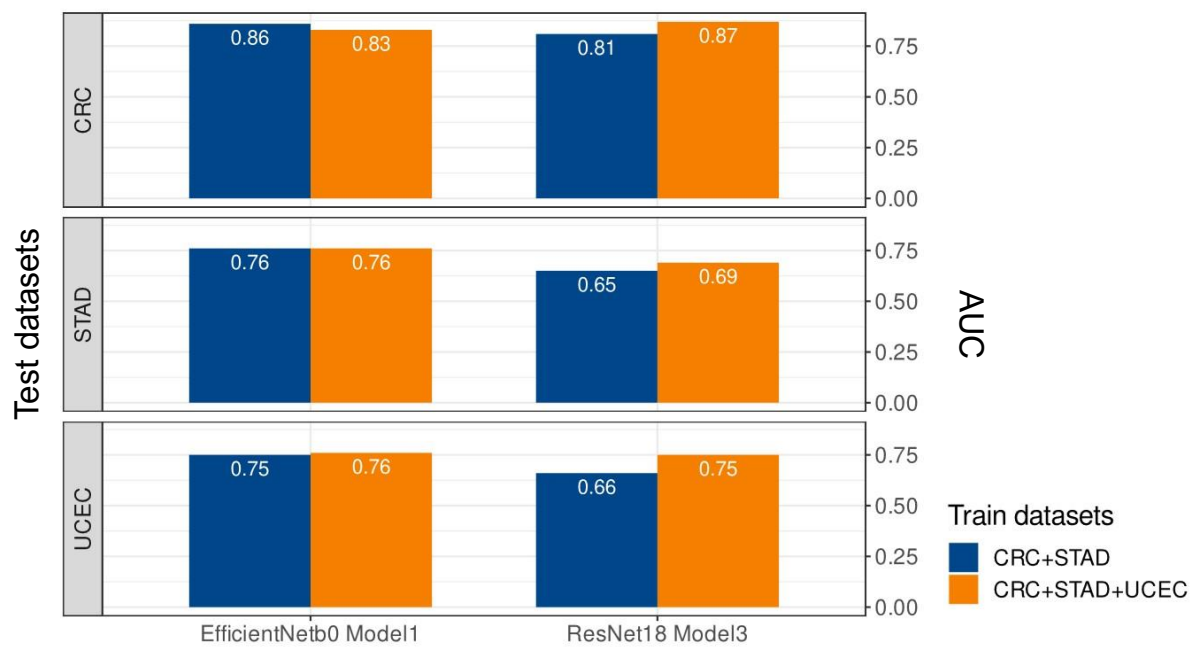

**S6 Fig. Comparing performances between two-tissue and three-tissue trained models.**

Supplement: S1 File — (ZIP) [file pone.0332034.s001.zip › Supporting_Information/S6_Fig.pdf]
