# Supplementary material for: Multi-cancer analysis of histopathologic MSI screening based on digital histology image
Source: PLoS One. 2025 Sep 15;20(9):e0332034. doi: 10.1371/journal.pone.0332034 (PMC12435642; doi:10.1371/journal.pone.0332034)
Supplement: Attachment — Submitted filename: Response_to_Reviewer_PlosOne.docx [file pone.0332034.s003.docx]

**Point-by-point Response to the Referee Comments**

**Reviewer: 1**

Comments to the Author

This is an important and relatively well performed study. However, there is no new innovation in this study and no outstanding technical or medical contribution. There have been many studies using TCGA dataset for MSI prediction on histologic images using CNN models. The models authors used were relatively outdated and do not contain any novelty.

**There is no external validation on any other public dataset such as CPTAC or PAIP. Authors would want to consider applying their models to other public datasets or other dataset from SNU.**

Response: We sincerely appreciate the reviewer's comment and agree to the reviewer's opinion regarding external validation. We have applied our models to public CPTAC datasets. The methodology and results of this comparison have been presented in the Materials and methods, Results sections, respectively.

**Materials and methods:**

“To perform external validation of our models, we utilized COAD and UCEC cohort datasets from CPTAC (Clinical Proteomic Tumor Analysis Consortium). A single WSI per patient was used for the validation, which comprised 18 MSI-H and 46 MSS patients from CPTAC-COAD, and 16 MSI-H and 58 MSS patients from CPTAC-UCEC.” (page 6, line 121-124)

“In the external validation assessment, evaluations were conducted on CPTAC-COAD and CPTAC-UCEC tissues using all available models, except the STAD-only trained model. This was due to the absence of STAD images in the CPTAC dataset.” (page 10, line 199-201)

**Results:**

“In the CPTAC datasets, 47,275 out of 101,811 tiles were identified as tumor regions from 64 CPTAC-COAD WSIs, while 43,318 out of 56,372 tiles were classified as tumor regions from 74 CPTAC-UCEC WSIs.” (page 12, line 236-238)

Table 2. The performance of our models (page 14 line 261 -262)

| **Datasets** | | **EfficientNetb0** | | | **ResNet18** | | | **VGG19** | | | **ConvNeXt** | | | **NAT** | | |
| --- | --- | --- | --- | --- | --- | --- | --- | --- | --- | --- | --- | --- | --- | --- | --- | --- |
| Train | Test | Model1 | Model2 | Model3 | Model1 | Model2 | Model3 | Model1 | Model2 | Model3 | Model1 | Model2 | Model3 | Model1 | Model2 | Model3 |
| CRC | CRC | 0.89 | 0.82 | **0.93** | 0.87 | 0.83 | 0.86 | 0.79 | 0.76 | 0.9 | 0.81 | 0.78 | 0.87 | 0.89 | 0.87 | 0.88 |
|  | STAD | 0.55 | 0.56 | 0.57 | 0.36 | 0.47 | 0.5 | 0.49 | 0.55 | 0.59 | 0.52 | 0.55 | 0.51 | 0.34 | 0.47 | 0.45 |
|  | UCEC | 0.57 | 0.56 | 0.6 | 0.58 | 0.53 | 0.6 | 0.59 | 0.54 | 0.58 | 0.55 | 0.53 | 0.54 | 0.60 | 0.53 | 0.54 |
|  | CPTAC_  COAD | 0.7 | 0.84 | 0.77 | 0.88 | 0.85 | 0.77 | 0.87 | 0.75 | 0.91 | 0.73 | 0.81 | 0.78 | 0.78 | 0.83 | 0.82 |
|  | CPTAC_  UCEC | 0.46 | 0.48 | 0.5 | 0.5 | 0.48 | 0.54 | 0.43 | 0.32 | 0.33 | 0.42 | 0.42 | 0.46 | 0.54 | 0.39 | 0.5 |
| STAD | CRC | 0.72 | 0.79 | 0.81 | 0.71 | 0.75 | 0.7 | 0.69 | 0.59 | 0.66 | 0.76 | 0.79 | 0.74 | 0.79 | 0.77 | 0.79 |
|  | STAD | **0.84** | 0.78 | 0.79 | 0.67 | 0.73 | 0.64 | 0.7 | 0.76 | 0.79 | 0.82 | 0.7 | **0.84** | 0.71 | 0.81 | 0.73 |
|  | UCEC | 0.57 | 0.67 | 0.62 | 0.65 | 0.61 | 0.6 | 0.59 | 0.63 | 0.67 | 0.64 | 0.65 | 0.66 | 0.64 | 0.7 | 0.67 |
| UCEC | CRC | 0.57 | 0.51 | 0.53 | 0.6 | 0.6 | 0.58 | 0.6 | 0.62 | 0.57 | 0.61 | 0.61 | 0.61 | 0.63 | 0.67 | 0.62 |
|  | STAD | 0.57 | 0.5 | 0.59 | 0.65 | 0.47 | 0.61 | 0.52 | 0.45 | 0.66 | 0.48 | 0.42 | 0.45 | 0.52 | 0.5 | 0.59 |
|  | UCEC | 0.69 | 0.65 | 0.63 | 0.65 | 0.68 | 0.6 | 0.66 | 0.65 | 0.65 | 0.67 | 0.64 | 0.68 | 0.62 | 0.62 | 0.62 |
|  | CPTAC_  COAD | 0.52 | 0.52 | 0.58 | 0.6 | 0.67 | 0.49 | 0.54 | 0.51 | 0.52 | 0.7 | 0.69 | 0.69 | 0.51 | 0.67 | 0.6 |
|  | CPTAC_  UCEC | 0.72 | 0.68 | 0.72 | 0.72 | 0.68 | 0.76 | 0.6 | 0.55 | 0.63 | 0.64 | 0.6 | 0.63 | 0.69 | 0.56 | 0.75 |
| Multi- tissue | CRC | 0.83 | 0.8 | 0.8 | 0.83 | 0.8 | 0.87 | 0.7 | 0.7 | 0.77 | 0.81 | 0.83 | 0.78 | 0.86 | 0.87 | 0.83 |
|  | STAD | 0.76 | 0.74 | 0.69 | 0.68 | 0.7 | 0.69 | 0.71 | 0.65 | 0.66 | 0.75 | 0.65 | 0.65 | 0.77 | 0.64 | 0.75 |
|  | UCEC | 0.76 | 0.74 | 0.7 | 0.61 | 0.78 | 0.75 | 0.67 | 0.67 | 0.74 | 0.72 | 0.72 | 0.75 | 0.77 | **0.79** | 0.76 |
|  | CPTAC_  COAD | 0.77 | 0.78 | 0.64 | 0.82 | 0.81 | 0.69 | 0.81 | 0.74 | 0.79 | 0.71 | 0.81 | 0.74 | 0.73 | 0.84 | 0.68 |
|  | CPTAC_  UCEC | 0.61 | 0.66 | 0.77 | 0.53 | 0.65 | 0.73 | 0.43 | 0.44 | 0.65 | 0.65 | 0.49 | 0.61 | 0.66 | 0.59 | 0.63 |

“Analysis of the CPTAC validation dataset shows that all models demonstrated similarly performance on the CPTAC-COAD dataset, comparable to when trained on TCGA-CRC and tested on CRC. ResNet18 Model 1 achieved 0.88 and VGG19 Model 3 reached 0.91, marking the highest performance. The UCEC validation data showed similar results to when models were trained on TCGA-UCEC and tested on UCEC. In multi-tissue testing, while all models demonstrated relatively consistent and stable performance for COAD data, they showed somewhat lower performance on UCEC data.” (page 15 line 277-283)

**Multiple instance learning or transformer-based learning can be considered for novel technical approach.**

Response: We appreciate the reviewer's suggestion. We have conducted additional analysis using two state-of-the-art models that incorporate attention mechanisms - ConvNeXt and NAT. The results of this comparison have been presented in the Results sections, respectively.

**Materials and methods:**

“We extended our evaluation by incorporating two recent high-performing vision architectures: ConvNeXt and NAT (Neighborhood Attention Transformer) [20, 21].” (page8, line 167-169)

Reference:

20. Zhuang Liu HM, Chao-Yuan Wu, Christoph Feichtenhofer, Trevor Darrell, Saining Xie. A ConvNet for the 2020s. arXiv. 2022;(2201.03545).

21. Ali Hassani SW, Jiachen Li, Shen Li, Humphrey Shi. Neighborhood Attention Transformer. arXiv. 2023;(2204.07143).

**Results**

“ConvNeXt showed mean AUC values of 0.82 in TCGA-CRC, 0.79 in TCGA-STAD, and 0.66 in TCGA-UCEC, and NAT showed mean AUC values of 0.88 in TCGA-CRC, 0.75 in TCGA-STAD, and 0.62 in TCGA-UCEC. EfficientNetb0 Model3 exhibited a highest performance of 0.93 for TCGA-CRC. EfficientNetb0 Model1 and ConvNeXt Model3 showed performance results of 0.84 for TCGA-STAD.” (page 12, line 246-251)

“For TCGA-CRC, ResNet18 Model3 and NAT model2 showed the highest performance with an AUC of 0.87, while the VGG19 models generally showed lower performance results compared to other models. In TCGA-STAD, NAT and EfficientNetb0 Model1 have the good performance with an AUC of 0.77 and 0.76, respectively.” (page 14, Line 267-271)

“NAT Model2 exhibits the best performance for TCGA-UCEC with an AUC of 0.79, which is higher than the highest AUC of 0.69 achieved by a model trained on that corresponding tissue type alone.” (page 14, line 274-page 15, line 276)

**Reviewer: 2**

Comments to the Author

This paper focuses on developing and evaluating deep learning models to detect microsatellite instability (MSI) using whole-slide images (WSI) from the TCGA dataset. The study targets three cancer types: colorectal cancer (CRC), stomach adenocarcinoma (STAD), and uterine corpus endometrial carcinoma (UCEC), utilizing convolutional neural networks (CNNs) like EfficientNet, ResNet18, and VGG19 to differentiate between high microsatellite instability (MSI-H) and microsatellite stable (MSS) tumor tiles. The results show that models perform best when tested on tissue types that match their training data, while performance drops when models are tested on different tissue types. The EfficientNet models outperformed the others, and the study found that while multi-tissue trained models sometimes improved performance, they did not always outperform single-tissue models. The paper highlights the challenge of balancing model generality and specificity in MSI detection. Future work aims to incorporate more advanced deep learning models and validate them with external datasets. There are two questions for the author and after doing minor changes, the paper can be accepted by Plos One.

1. **Expand the Discussion on CNN Architectures**: The authors should provide a more in-depth discussion on the impact of different CNN architectures on model accuracy. Specifically, it would be beneficial to explain how the structural differences between EfficientNet, ResNet18, and VGG19 affect the model's ability to detect MSI. Including insights on how these architectures handle variations in histopathological features and why one may outperform the others in certain tissue types would enhance the technical understanding.

Response: We appreciate the reviewer's suggestion. We have revised the Discussion section as follows. (page19, line384-page 21, line 424)

“In our study, we employed three distinct CNN architectures. VGG19 features a uniform structure with nineteen layers and 3×3 convolution filters, demonstrating exceptional capability in extracting detailed visual features. While its hierarchical feature learning excels at capturing subtle tissue patterns, the model faces challenges with gradient vanishing due to its deep structure and high computational costs stemming from its 140 million parameters [35]. ResNet18 addresses deep structure's gradient vanishing problem by introducing shortcut connections. Despite its relatively shallow 18-layer structure, its residual learning approach enables efficient learning of complex tissue patterns while reliably preserving important feature information [36]. EfficientNetb0 achieves high accuracy and efficiency through compound scaling methods that automatically adjust network width, depth, and resolution, combined with Neural Architecture Search. While particularly adept at processing various scales of features in histopathological images, it presents implementation challenges due to its complex architecture and risks overfitting on smaller datasets [37]. To leverage the advantages of recent attention mechanisms, we evaluated two state-of-the-art models. ConvNeXt incorporates transformer design principles into CNN architecture, utilizing 7×7 kernels instead of traditional 3×3 kernels, expanding channel capacity, and introducing Layer Normalization and Depthwise Convolution. However, this model risks overfitting on small datasets and may exhibit unstable transfer learning performance across different domains [38]. NAT effectively combines attention mechanisms with hierarchical processing, achieving a balance between local context preservation and computational efficiency while successfully implementing CNN strengths such as locality, translation equivariance, and hierarchical feature representation. However, its complex structure makes model tuning and optimization challenging for specific tasks, and performance can vary significantly depending on task characteristics [39]. This comprehensive analysis revealed distinct performance patterns across models, varying significantly with cancer type characteristics and transfer learning strategies.

In the performance analysis of multi-tissue training, distinctive patterns emerged across different validation datasets. EfficientNetb0 Model 1 achieved the highest and most stable performance on internal TCGA datasets, with a mean accuracy of 0.78 (SD=0.04). In a broader evaluation including both internal TCGA and external CPTAC datasets, NAT Model 1 demonstrated the highest overall performance, achieving a mean accuracy of 0.76 (SD=0.072), while EfficientNetb0 Model 2 exhibited the most robust generalization capability, with a mean accuracy of 0.74 (SD=0.05).

These performance variations reflect the fundamental architectural differences of each model. The compound scaling methodology central to EfficientNetb0 effectively adjusts network depth, width, and resolution, enabling the capture of multi-scale features in medical images. This adaptability likely contributed to its stable performance across diverse tissue types. Meanwhile, the neighbor attention mechanism in NAT’s architecture demonstrated a strong capability in integrating local features with global contextual information. This architectural advantage allowed for consistent extraction of crucial visual features from pathological images, even those collected from institutions with varying characteristics, contributing to NAT's strong performance across diverse datasets.”

2. **Add Experiments to Investigate Generalization Issues**: The authors should conduct additional experiments to explore the underlying reasons for the model's limited generalization across tissue types. Investigating the role of tissue-specific features, differences in tumor microenvironments, or variations in data distribution could provide valuable insights.

These experiments would help identify the factors that hinder the model's ability to generalize and offer potential solutions to improve its performance in cross-tissue predictions.

Response: We appreciate the reviewer's comments. We have revised the Discussion section as follows. (page 18 line 357-page 19 line 383)

“Colorectal cancer, gastric cancer, and endometrial cancer each exhibit distinct molecular characteristics [26-29] and tumor microenvironments (TME) [30]. According to Tumor-Infiltrating Lymphocyte (TIL) mapping studies based on H&E images from TCGA samples, gastric cancer shows the highest TIL ratio at approximately 14.6% and exhibits histological features of immune response across more extensive regions compared to other cancer types [31]. Analysis of matrisome gene expression patterns, a key component of TME, across these three cancers reveals that colorectal and gastric cancers share similar expression patterns forming a single cluster, while endometrial cancer shows unique matrisome transcription factor regulation patterns distinct from the other cancer types [32]. Furthermore, TCGA cohort samples display diverse clinical characteristics, and tumor histological characteristics may vary due to biological differences among the medical centers where patients received treatment [33].

MSI detection across multiple cancer types presents several challenges. While MSI classification in a single cancer type involves binary classification between MSI and non-MSI within that cancer type, multi-tissue trained model classification requires the model to understand and learn diverse manifestations of MSI across different cancer types, resulting in a more complex decision-making process. These models face technical difficulties in distinguishing cancer-type differences and MSI status. Additionally, MSI-related features often appear as weak signals overshadowed by the dominant characteristics of each cancer type, making it challenging to identify subtle MSI patterns. To address these challenges, we evaluated the performance of various model architectures with different characteristics. However, the performance of multi-cancer model did not surpass that of single-cancer models, likely due to the complexities of multi-domain learning and increased task difficulty. To overcome these limitations, we suggest that future work should focus on developing specialized architectures that employ domain adaptation techniques (such as Domain Adversarial Neural Networks - DANN) to normalize cancer-type-specific histological features and align MSI feature distributions across cancer types [34].”

References

26. Cancer Genome Atlas N. Comprehensive molecular characterization of human colon and rectal cancer. Nature. 2012;487(7407):330-7. Epub 20120718. doi: 10.1038/nature11252. PubMed PMID: 22810696; PubMed Central PMCID: PMCPMC3401966.

27. Cancer Genome Atlas Research N. Comprehensive molecular characterization of gastric adenocarcinoma. Nature. 2014;513(7517):202-9. Epub 20140723. doi: 10.1038/nature13480. PubMed PMID: 25079317; PubMed Central PMCID: PMCPMC4170219.

28. Cancer Genome Atlas Research N, Kandoth C, Schultz N, Cherniack AD, Akbani R, Liu Y, et al. Integrated genomic characterization of endometrial carcinoma. Nature. 2013;497(7447):67-73. doi: 10.1038/nature12113. PubMed PMID: 23636398; PubMed Central PMCID: PMCPMC3704730.

29. Liu Y, Sethi NS, Hinoue T, Schneider BG, Cherniack AD, Sanchez-Vega F, et al. Comparative Molecular Analysis of Gastrointestinal Adenocarcinomas. Cancer Cell. 2018;33(4):721-35 e8. Epub 20180402. doi: 10.1016/j.ccell.2018.03.010. PubMed PMID: 29622466; PubMed Central PMCID: PMCPMC5966039.

30. Thorsson V, Gibbs DL, Brown SD, Wolf D, Bortone DS, Ou Yang TH, et al. The Immune Landscape of Cancer. Immunity. 2018;48(4):812-30 e14. Epub 20180405. doi: 10.1016/j.immuni.2018.03.023. PubMed PMID: 29628290; PubMed Central PMCID: PMCPMC5982584.

31. Saltz J, Gupta R, Hou L, Kurc T, Singh P, Nguyen V, et al. Spatial Organization and Molecular Correlation of Tumor-Infiltrating Lymphocytes Using Deep Learning on Pathology Images. Cell Rep. 2018;23(1):181-93 e7. doi: 10.1016/j.celrep.2018.03.086. PubMed PMID: 29617659; PubMed Central PMCID: PMCPMC5943714.

32. Izzi V, Lakkala J, Devarajan R, Kaariainen A, Koivunen J, Heljasvaara R, et al. Pan-Cancer analysis of the expression and regulation of matrisome genes across 32 tumor types. Matrix Biol Plus. 2019;1:100004. Epub 20190406. doi: 10.1016/j.mbplus.2019.04.001. PubMed PMID: 33543003; PubMed Central PMCID: PMCPMC7852311.

33. Howard FM, Dolezal J, Kochanny S, Schulte J, Chen H, Heij L, et al. The impact of site-specific digital histology signatures on deep learning model accuracy and bias. Nat Commun. 2021;12(1):4423. Epub 20210720. doi: 10.1038/s41467-021-24698-1. PubMed PMID: 34285218; PubMed Central PMCID: PMCPMC8292530.

34. Guan H, Liu M. Domain Adaptation for Medical Image Analysis: A Survey. IEEE Trans Biomed Eng. 2022;69(3):1173-85. Epub 20220218. doi: 10.1109/TBME.2021.3117407. PubMed PMID: 34606445; PubMed Central PMCID: PMCPMC9011180.
