# Supplementary material for: Multi-cancer analysis of histopathologic MSI screening based on digital histology image
Source: PLoS One. 2025 Sep 15;20(9):e0332034. doi: 10.1371/journal.pone.0332034 (PMC12435642; doi:10.1371/journal.pone.0332034)
Supplement: Attachment — Submitted filename: R2_Response_to_Reviewer_PlosOne.docx [file pone.0332034.s004.docx]

**Point-by-point Response to the Referee Comments**

**Reviewer: 1**

Comments to the Author

1. Which of these features does the model rank highest when distinguishing MSI-H from MSS? Is it possible to use interpretability tools such as LIME or SHAP to visualize and understand these features? I suggest the author writes more to extend the Section “Geographic visualization and comparative analysis of MSI prediction scores”**.**

Response: We sincerely appreciate the reviewer's comment To understand which features the model ranks highest when distinguishing MSI-H from MSS, we pathologically analyzed regions with high probability values and regions with low probability values in the prediction heatmap. Based on these findings, we have expanded the section "Geographic visualization and comparative analysis of MSI prediction scores.

**Results:**

**Geographic visualization and comparative analysis of MSI prediction scores**

“To understand which features the model ranks highest when distinguishing MSI-H from MSS, we pathologically analyzed regions with high probability values and regions with low probability values in the prediction heatmap. The results revealed that areas highly scored as MSI-H predominantly exhibited features of poorly differentiated carcinoma and high amounts of tumor infiltrating lymphocytes. This aligns with the histological characteristics of MSI-H known from existing pathological research. Conversely, tissue regions highly scored as MSS displayed features of well to moderately differentiated carcinoma (Fig 4).” (page 17 line 307-313)

**Figure legend:**

“Fig 4. Comparative examples of MSI prediction patterns and tissue morphology. The prediction heatmaps ( a and c) display results generated using an EfficientNet Model1 architecture with multi-tissue training. These maps show predicted microsatellite instability status across tissue samples, with corresponding H&E histology images (b and d) revealing the actual tissue morphology from regions marked by white boxes. A and B represent colorectal cancer and stomach cancer, respectively, with results showing microsatellite instability high (a, MSI scores: 0.85 and 0.88) and microsatellite stable (c, MSI scores: 0.18 and 0.08) status.”(page18 line323- line329)

2. I suggest that the author could Implement robust cross-validation techniques to ensure the models are not overfitting, such as k-fold cross-validation or stratified splits based on cancer types.

Response: We appreciate the reviewer's suggestion regarding robust cross-validation techniques. We would like to clarify that our study already implemented 5-fold cross-validation to ensure our models were not overfitting, as illustrated in Supplementary Figure S1. Furthermore, as shown in Supplementary Figure S3, we maintained balanced distributions of MSI-H and MSS cases across all cancer types (TCGA-CRC, TCGA-STAD, and TCGA-UCEC) in both training and testing sets, as well as within each fold of cross-validation. We created separate models for each cancer type, as well as a combined model trained on data from all three cancer types. For the multi-tissue model, we ensured uniform distribution across the three cancer types to address tissue-specific heterogeneity, preventing any single cancer type from dominating the learning process. We have added a detailed explanation of this cross-validation and sampling strategy in the methods section.

**Materials and methods:**

**A MSI tile classification model development**

To ensure robust model performance and prevent overfitting, we implemented a comprehensive cross-validation strategy. For the initial data split, we allocated 80% of the patients to the training set and 20% to the testing set, maintaining the proportion of MSI-H and MSS patient consistent across both sets for each cancer type (TCGA-CRC, TCGA-STAD, and TCGA-UCEC) (Table 1). Within the training set, we further divided the data into 5 patient-level folds for 5-fold cross-validation for hyperparameter tuning and model selection (S1B Fig). For every fold, the same number of patches was randomly selected for each class (S3 Fig). We created separate models for each cancer type as well as a combined model using data from all three tissue types. For the combined model, we maintained uniform distributions across cancer types to prevent any single cancer type from dominating the learning process. (page 8 line 163 - 172)

3. How do the diagnostic accuracies of deep learning models compare with those of traditional methods—such as PCR and immunohistochemistry—in terms of sensitivity, specificity, and overall diagnostic yield?

Response: We appreciate the reviewer's comment and agree to the reviewer's opinion regarding comparison with other methods. However, we would like to address several important methodological limitations in our study. Regarding comparison with PCR, our deep learning model was trained using PCR results as the gold standard. Therefore, directly comparing the diagnostic accuracy of PCR with our deep learning model is not methodologically appropriate. Although we cannot claim that our model is superior to PCR results, we observe that the AUC values of our models show substantial concordance with PCR results, suggesting our model demonstrates potential diagnostic utility in clinical settings. Regarding comparison with immunohistochemistry, our research dataset does not include immunohistochemistry test results, making it currently impossible to compare this traditional method with our deep learning model. Given these limitations, we rigorously evaluated the performance of our deep learning model through various approaches. We experimented with several deep learning architectures and used k-fold cross-validation during model training to prevent overfitting. Additionally, we assessed the generalization performance of our model using independent validation and test datasets that were not utilized during training datasets. We have additionally included comprehensive performance metrics (accuracy, precision, recall, specificity, and F1 scores) for all models in S7 Table to provide a detailed evaluation. These methods ensured the robustness and reliability of our model. In future research, we plan to conduct direct comparisons between our deep learning model and various other diagnostic methods, including immunohistochemistry. These limitations and our future plans have been described in the discussion section of our manuscript.

**Results**

**MSI-H classification model evaluation in slide level**

Detailed model performance metrics, including accuracy, precision, recall, specificity, and F1 scores for all evaluated models, can be found in S7 Table.(page 16 line284-285)

**Discussion**

“The dataset utilized in this study does not include immunohistochemistry test results, another diagnostic method for MSI, making it currently impossible to compare these immunohistochemistry results with our deep learning model. Recognizing these limitations, in future research, we plan to establish an independent validation cohort to conduct direct comparisons between our deep learning model and various other diagnostic methods, including immunohistochemistry.” (page 23, line 458-463)
