# Supplementary material for: Multi-cancer analysis of histopathologic MSI screening based on digital histology image
Source: PLoS One. 2025 Sep 15;20(9):e0332034. doi: 10.1371/journal.pone.0332034 (PMC12435642; doi:10.1371/journal.pone.0332034)
Supplement: Attachment — Submitted filename: R3_Response_to_Reviewer_PlosOne.docx [file pone.0332034.s005.docx]

**Point-by-point Response to the Referee Comments**

**Reviewer: 2**

Comments to the Author

This is a well-structured and methodologically sound study addressing a clinically relevant problem: using deep learning to predict microsatellite instability (MSI) from standard histology slides across multiple cancer types. The paper is clearly written, the experiments are comprehensive, and the discussion is thoughtful and balanced. The authors systematically compare models trained on single cancer types, evaluate their cross-cancer generalizability, and test a combined multi-cancer model. The inclusion of an external validation cohort (CPTAC) significantly strengthens the findings. The conclusion that multi-tissue models can improve performance for some cancers (UCEC) while not for others (CRC, STAD) is a nuanced and important contribution to the field.

The manuscript is of high quality and suitable for publication, pending minor revisions to enhance clarity and address a few key points.

Throughout: The term "hypterparameters" is used several times (e.g., line 151, 154, 184); it should be "hyperparameters".
Line 90: "staomach" should be "stomach". (Also seen in Figure 1).
Line 101: "publicy" should be "publicly".
Line 149: "emplolyed" should be "employed".
Line 174: "arcituecture" should be "architecture".
Line 189: "cutomized models" should be "customized models".
Line 212: "demonstarate" should be "demonstrate". "calssifier" should be "classifier". "chracteriestic" should be "characteristic".
Line 221: "a overall accuracy" should be "an overall accuracy".
Line 237: "classifcation model" should be "classification model".
Line 269 (Table 2 Title): "performnace" should be "performance".
Line 351: "We thougth that is may have" could be rephrased for clarity, e.g., "We thought that this might have..."

Response: Thank you for pointing out the typographical errors. We have carefully reviewed the manuscript and corrected all identified typos.

**Reviewer: 3**

Comments to the Author

Thanks for making these revisions. I am satisfied with the current version. The authors may consider including relevant citations on image-guided cancer research to strengthen the study's background, such as:"

*DOI: 10.1016/j.cpsurg.2025.101819*

*DOI: 10.1016/j.cpsurg.2025.101833*

*DOI: 10.1016/j.cpsurg.2025.101817*

*DOI: 10.1016/j.cpsurg.2024.101640*

Response: We thank the reviewer for the suggestion to include additional citations. Following this recommendation, we have added relevant content on image-based deep learning approaches for cancer research in the introduction, including several of the references to strengthen the study's background.

**Introduction:**

“Given these limitations, there is a growing need for more accessible and efficient MSI detection methods.

Over the past decade, image analysis with deep learning (DL) has drastically improved, leading to the development of numerous models that assist clinicians in formulating personalized cancer treatment strategies. These predictive models have leveraged diverse imaging modalities, including, magnetic resonance imaging (MRI) for tumor staging and treatment planning [7], and CT/PET imaging for predicting treatment response in lung cancer [8], and immunohistochemistry (IHC) images for genetic profile prediction and treatment response assessment [9]. Of particular importance, hematoxylin and eosin (H&E) stained slide images exhibit how various genetic alterations manifest as distinctive patterns in histopathological images [10]. Predictive models based on these genetic profiles enable the prediction of immunotherapy response or survival analysis. For example, MSI-H colorectal cancer histopathologic images present more tumor-infiltrating lymphocytes, mucinous differentiation, medullary-like morphology, or lack of necrosis [11, 12]. Therefore, an alternative screening method using DL models to analyze MSI features from H&E slide images may provide a more rapid and cost-effective diagnosis method compared to traditional approaches. Such an approach could potentially enhance patient outcomes and improve clinical workflows.” (page 3 line 60 – page 4 line 77)

**Reference:**

7.	Zhang L, Wang K, Xiang P. Analysis of the clinical tumor stage and survival prognosis of rectal cancer patients on the basis of deep learning and imaging characteristics: An observational study. Curr Probl Surg. 2025;69:101817. Epub 20250529. doi: 10.1016/j.cpsurg.2025.101817. PubMed PMID: 40716857.

8.	Guzman Gomez R, Lopez Lopez G, Alvarado VM, Lopez Lopez F, Esqueda Cisneros E, Lopez Moreno H. Deep Learning Approaches for Automated Prediction of Treatment Response in Non-Small-Cell Lung Cancer Patients Based on CT and PET Imaging. Tomography. 2025;11(7). Epub 20250630. doi: 10.3390/tomography11070078. PubMed PMID: 40710896; PubMed Central PMCID: PMCPMC12298732.

9.	Brattoli B, Mostafavi M, Lee T, Jung W, Ryu J, Park S, et al. A universal immunohistochemistry analyzer for generalizing AI-driven assessment of immunohistochemistry across immunostains and cancer types. NPJ Precis Oncol. 2024;8(1):277. Epub 20241203. doi: 10.1038/s41698-024-00770-z. PubMed PMID: 39627299; PubMed Central PMCID: PMCPMC11615360
